# Complete mitochondrial genome of sculptured slipper lobster *Parribacus antarcticus* (Lund, 1793)

**DOI:** 10.1080/23802359.2019.1627934

**Published:** 2019-07-11

**Authors:** Mingqiu Yang, Hongtao Liu, Yugui He

**Affiliations:** Hainan Provincial Key Laboratory of Tropical Maricultural Technologies, Hainan Academy of Ocean and Fisheries Sciences, Haikou, China

**Keywords:** Mitochondrial genome, *Parribacus antarcticus*, phylogenetic analysis

## Abstract

We first determined and characterized the complete mitochondrial genome of *Parribacus antarcticus*. It is 15,806 bp long and consists of 22 tRNA, 2 rRNA, 13 protein-coding genes (PCGs), and 1 control region. The nucleotide composition is significantly biased with AT contents of 69.3%. Five PCGs used an unusual initiation codon, and nine PCGs were terminated with an incomplete or abnormal stop codon. Three microsatellites were identified and located in the *ND4* gene and D-loop region. Phylogenetic tree showed that P. antarcticus was first clustered with Ibacus ciliatus and Ibacus alticrenatus, which is consistent with the expected phylogenetic relationship.

*Parribacus antarcticus*, commonly known as sculptured slipper lobster or sculptured mitten lobsters, is an edible economic species in the family Scyllaridae, Achelata, which is distributed from Florida to north-east Brazil in the Atlantic and from the east and south-east Africa to Hawaii and Polynesia in the Indo-West Pacific region (Palero et al. 2014). It mainly inhabits coral reefs or deep-sea reefs and shelters solitarily (Sharp et al. 2007). From the mid-19th century, several studies have been concentrated on the biology of *P. antarcticus*, including reproduction (Matthews 1954a, 1954b), larval development (Ikeda et al. 2011; Palero et al. 2014), ecological observation (Sharp et al. 2007) and new distribution (Wahyudin et al. 2017; Freitas and Wirtz 2018).

The samples were obtained from Huanqiu wharf of Wenchang, Hainan province, China (19°33′51.12″N, 110°49′27.98″E), and stored in the marine crustacean specimen room in Qionghai research base of Hainan Academy of Ocean and Fisheries Sciences for reference, Muscle samples of *P. antarcticus* were preserved in absolute ethanol for total DNA extraction.

The complete mitogenome of *P. antarcticus* is 15,806 bp in length (GenBank Accession No. MK783264). The base content was 34.5% A, 12.1% G, 34.8% T, and 18.6% C. The 69.3% of (A + T) showed great preference to AT. The mitogenome sequence consists of 22 tRNA genes, 2 rRNA genes, 13 protein-coding genes (PCGs), and 1 control region (D-loop). Four PCGs (*ND1*, *ND4*, *ND4L*, and *ND5*), eight tRNA genes and two rRNA genes were located on the light strand, the others were encoded by the heavy strand.

The 22 tRNA genes in mitogenome of *P. antarcticus* vary in size from 63 to 72 bp. There are two types of *tRNA-Leu* identified with the codons TAA and TAG, which always were used as stop codons. The 12S rRNA is 863 bp and located between *tRNA-Val* and D-loop, and the 16S rRNA is 1322 bp, located between *tRNA-Val* and *tRNA-Leu*. Except for eight PCGs using the normal ATN as the start codon, the others use an unusual initiation codon (*ND1* and *ND4L* use TTA; *COX1* uses ACG; *ND4* uses CAC; *ND5* uses CTA). Simultaneously, 9 PCGs were terminated with an incomplete or unusual codon in addition to 4 PCGs genes using the normal stop codon TAA. Among the 9 PCGs, *COX1*, *ND2*, and *ND3* use a single T; *ND4* uses AT; *COX2* uses TC; *CYTB* uses TG; *ND1* and *ND5* use CAC; *ND4L* uses CAT. The control region is 849 bp, located between 12S rRNA and *tRNA-Ile*. Interestingly, we identified three microsatellites (SSR) in *P. antarcticus* mitogenome using MISA. A (TA)_6_ SSR is located in the codon region of *ND4* genes, another (TA)_6_ SSR and an (A)_10_ SSR are both situated in the control region.

Based on nucleotide sequences of 16 Achelata species mitogenome available in the GenBank, a phylogenetic analysis was carried out to investigate the evolution position of *P. antarcticus* using the maximum–likelihood (ML) method with 1000 bootstrap replicates. The result (Figure 1) showed that *P. antarcticus* was first cluster to *Ibacus ciliatus* and *Ibacus alticrenatus*, which is consistent with the expected phylogenetic relationship.

**Figure 1.**
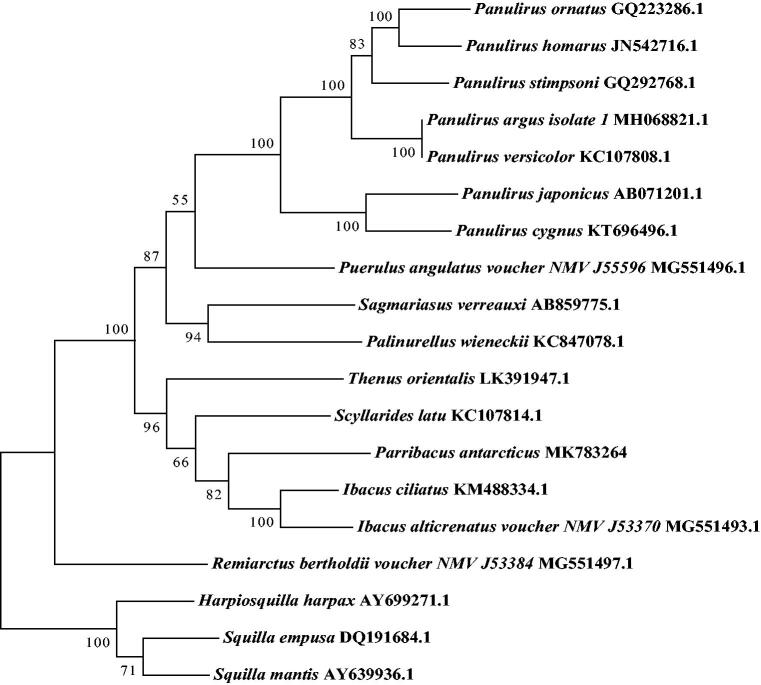
Phylogenetic tree of the complete mitogenome of 16 species in Achelata. *Harpiosquilla harpax*, *Squilla empuse* and *Squilla mantis* were used as outgroups.
